# Metabolomics Signatures of a Respiratory Tract Infection During an Altitude Training Camp in Elite Rowers

**DOI:** 10.3390/metabo15060408

**Published:** 2025-06-17

**Authors:** Félix Boudry, Fabienne Durand, Corentine Goossens

**Affiliations:** 1 Espace Dev, Université de Perpignan Via Domitia, 66860 Perpignan, France; 2 UMR Espace Dev (228), Université Montpellier, IRD, 34093 Montpellier, France; 3 CNRS-EPHE-UPVD, UAR3278, CRIOBE, 66860 Perpignan, France

**Keywords:** metabolomics, PLS-DA, respiratory pathology, altitude training, athlete health, NMR

## Abstract

**Background**: Respiratory pathologies, such as COVID-19 and bronchitis, pose significant challenges for high-level athletes, particularly during demanding altitude training camps. Metabolomics offers a promising approach for early detection of such pathologies, potentially minimizing their impact on performance. This study investigates the metabolic differences between athletes with and without respiratory illnesses during an altitude training camp using urine samples and multivariate analysis. **Methods**: Twenty-seven elite rowers (15 males, 12 females) participated in a 12-day altitude training camp at 1850 m. Urine samples were collected daily, with nine athletes developing respiratory pathologies (8 COVID-19, 1 bronchitis). Nuclear Magnetic Resonance spectroscopy was used to analyze the samples, followed by data processing with Principal Component Analysis (PCA) and Partial Least Squares Discriminant Analysis (PLS-DA), allowing to use Variable Importance in Projection (VIP) scores to identify key metabolites contributing to group separation. **Results**: The PLS-DA model for respiratory illness showed good performance (R^2^ = 0.89, Q^2^ = 0.35, *p* < 0.05). Models for altitude training achieved higher predictive power (Q^2^ = 0.51 and 0.72, respectively). Metabolites kynurenine, *N*-methylnicotinamide, pyroglutamate, propionate, *N*-formyltryptophan, tryptophan and glucose were significantly highlighted in case of respiratory illness while trigonelline, 3-hydroxyphenylacetate, glutamate, creatine, citrate, urea, o-hydroxyhippurate, creatinine, hippurate and alanine were correlated to effort in altitude. This distinction confirms that respiratory illness induces a unique metabolic profile, clearly separable from hypoxia and training-induced adaptations. **Conclusions**: This study highlights the utility of metabolomics in identifying biomarkers of respiratory pathologies in athletes during altitude training, offering the potential for improved monitoring and intervention strategies. These findings could enhance athlete health management, reducing the impact of illness on performance during critical training periods. Further research with larger cohorts is warranted to confirm these results and explore targeted interventions.

## 1. Introduction

Rowing is a discipline that combines endurance and power, requiring both high static and dynamic capacities [1]. In elite athletes, rowing performance largely relies on the oxygen uptake [2]. In the pursuit of improved respiratory capacity and oxygen consumption, high-level athletes and trainers searched for efficient training methods. Altitude training has become a fundamental component of athletic preparation, by promoting a range of physiological adaptations that enhance oxygen transport and utilization, especially in endurance disciplines [3]. These adaptations result from mechanisms associated with chronic hypoxic acclimatization, further amplified by the physiological stress imposed by training under hypoxic conditions [4]. This practice is particularly recognized for its beneficial effects on endurance performance, through the physiological adaptations induced by exposure to hypoxia via altitude training camp [5]. Altitude camps are strategically scheduled to maximize performance gains, either early in the season to build a solid fitness foundation or shortly before competitions to elicit specific adaptations.

However, while these adaptations may enhance performance, the significant demands imposed by altitude training can also affect the respiratory system, which, in elite endurance athletes, may not always be fully adapted to meet the increased requirements of training and competition [6]. Furthermore, hypoxia associated with intense training can lead to immune depression, increasing the susceptibility of athletes to infections, particularly of the respiratory tract [7]. This risk is exacerbated by cumulative stress factors commonly encountered during altitude training camps, such as sleep deprivation, moderate overtraining and challenging environmental conditions. The respiratory system is especially vulnerable as it is directly exposed to the environment and unusual training conditions [8]. Together, these factors may impair immune function and make athletes more vulnerable to illness during these critical preparation periods [9]. The onset of respiratory illnesses during these key moments can disrupt training plans and compromise performance outcomes [10].

A comprehensive understanding of the mechanisms linking pathological responses and physiological adaptations to hypoxic training may offer significant value. Such knowledge could enable more precise training planification and allow for the calibration of hypoxic stress to optimize physiological adaptations while minimizing the risk of inducing or exacerbating pathological conditions [11]. These adaptations are ultimately directed toward enhanced athletic performance and represent a tangible interface between physiology processes and the athlete phenotype, a biological dimension that can be explored through metabolomic approaches.

Metabolomics is the comprehensive and high-throughput analysis of low-molecular-weight metabolites within a biological system, providing a dynamic snapshot of the organism’s physiological or pathological state at a specific time point [12]. Because metabolites are the downstream products of cellular regulatory processes, their profiling provides a sensitive readout of biochemical activity and, by extension, the functional state of cells, tissues or organisms. This makes metabolomics particularly powerful for linking external environmental conditions, lifestyle factors or physiological stressors to molecular and systemic responses [13,14]. Its sensitivity to detect early and subtle metabolic shifts allows the identification of potential biomarkers or predictors of physiological adaptation before phenotypic changes become evident [15]. This capacity is particularly valuable in contexts such as exercise physiology, where metabolic flexibility and adaptation are central concerns [16,17]. If other methods such as mass spectrometry are available and more sensitive to metabolite concentrations, proton Nuclear Magnetic Resonance (^1^H NMR) spectroscopy-based metabolomics is commonly employed in this context, offering highly reproducible protocols for metabolite identification [18]. Following this purpose, using urine samples with ^1^H NMR offers several advantages: it is non-invasive, readily obtainable in large volumes and requires minimal preparation. [19,20], characterizing respiratory disease conditions [21,22] and tracking training responses, both under normal conditions [23,24,25] and in specific conditions such as hypoxia [26,27] underscoring its relevance in integrative and personalized physiology.

The objective of the present study is to characterize the specific metabolic alterations associated with respiratory illnesses that arise during an altitude training camp in elite rowers, and to distinguish these changes from those induced by physiological adaptations to sustained hypoxic training. While previous studies have explored metabolic responses to either respiratory disease or altitude exposure independently, few have investigated their overlap in a real-world, high-performance athletic setting. By applying ¹H NMR-based urinary metabolomics, this study aims to identify distinct metabolite signatures and disrupted metabolic pathways specifically linked to respiratory pathologies, while rigorously accounting for the confounding effects of hypoxia and intensive training loads. This approach not only enhances our understanding of the metabolic impact of respiratory conditions in elite athletes but also contributes to the development of non-invasive diagnostic strategies for early detection and monitoring of illness in high-altitude sports environments.

## 2. Materials and Methods

### 2.1. Participants

Twenty-seven high-level rowers (15 men and 12 women, Table 1) from the French rowing federation participated in this study. All were taking part in a 12-day altitude training camp at 1850 m of altitude organized by the French rowing federation. No external athletes were included and no interventions on the training or training camp plan were made by any of the investigators. Participants resided at sea level, with no stays above 500 m in the 3 months preceding the protocol introduction. They trained at least 8 h per week in the previous 5 years and had no respiratory diseases, cardiovascular diseases or tobacco use. No specific diet was followed during the training camp for the purpose of this study. All athletes followed a meal plan provided for the whole team by a professional nutritionist from the training camp center.

Approval for this study was obtained from an ethics committee (CERSTAP IRB00012476-2023-17-10-271). All participants gave their written informed consent.

### 2.2. Experimental Design

Participants took part in their federal altitude training camp as usual. There was no intervention in training. Urine samples were collected from all participants throughout the entire duration of the training camp, each morning immediately upon waking and before breakfast. To ensure the integrity of the samples, they were immediately frozen and stored at −80 °C until analysis. A total of 292 urine samples were collected. The health status (symptoms and antigenic test for COVID-19) of all athletes was assessed each morning by discussion with the medical staff. Of the 27 participants, 9 athletes (4 men and 5 women) developed respiratory illnesses between day 5 and day 12 of the training camp. These cases, 8 COVID-19 infections and 1 bronchitis, were identified incidentally during routine medical monitoring conducted by the team’s medical staff. Monitoring included COVID-19 screening and clinical evaluation in response to symptom occurrence. Although individual symptom profiles were not systematically recorded, all cases presented with classical, respiratory symptoms consistent with upper respiratory tract infection (sore throat, fatigue, mild fever), and none required hospitalization or specific medical intervention beyond routine care. No other respiratory infections were diagnosed or suspected during the camp.

### 2.3. Sample ^1^H NMR Acquisition and Data Processing

Urine samples were thawed at room temperature just before analysis and were vortexed for homogenization. An amount of 600 µL of urine was mixed with 150 µL of phosphate buffer (50 mM, pH 7.2) in D2O containing 0.002% of 3-(trimethylsilyl)propionic-2,2,3,3-d4 acid sodium salt (TMSP) (Eurisotop, Saint-Aubin, France). The mixture was vortexed and centrifuged at 14,000 rpm for 15 min. A volume of 550 µL of the supernatant was transferred into a 5 mm Norell 500-7 ^1^H NMR tube.

Urine samples for ^1^H NMR analysis were randomized before acquisition and ^1^H NMR spectra were acquired using a 500 MHz ECZR JEOL spectrometer (Tokyo, Japan). For each spectrum, a presaturation of the water peak was performed and a total of 32 scans was collected in 65K data points over a spectral width of 12 ppm, using a relaxation delay of 10.64 s, at 25 °C. Shims criteria were optimized based on the TMSP half-peak width, which was set at a maximum of 1Hz and its variability that of the signal-over-noise ratio across all samples did not exceed 10%. All ^1^H NMR analyses were performed on the bio2mar MSXM platform at the University of Perpignan (https://bio2mar-msxm.univ-perp.fr/ (accessed on 13 June 2025)).

An exponential function corresponding to a line broadening of 0.3 Hz was applied on all free induction decay signals prior to Fourier transformation. The resulting spectra were then processed using NMRProcFlow software (v1.4) [28] for baseline correction, phase adjustment, and chemical shift referencing to the TMSP internal standard. Spectra were then binned into segments of variable size using the “intelligent bucketing” function, and the area under each bin was calculated for each bucket.

### 2.4. Data Analysis and Statistics

Values are presented in “mean ± standard deviation (SD)” format. Statistical analysis was conducted using R (v4.4.2) [29] and the significance level was fixed at 0.05.

Data were normalized by constant sum and scaled (mean-centered and divided by the SD of each variable). To explore the variance in the metabolomic data and identify potential grouping patterns, a Principal Component Analysis (PCA) was conducted as an unsupervised method. PCA reduces the dimensionality of the dataset by summarizing it into Principal Component (PC), which represents the directions of maximum variance. After that, a Partial Least-Square Discriminant Analysis (PLS-DA) was conducted to create a supervised model that aimed to maximize class separation, by using labels, while identifying the most relevant features contributing to that separation based on the Variable Importance in Projection (VIP). Each identified bucket (each one corresponding to a VIP) was, if possible, associated with a metabolite based on the current literature and on the database available in Chenomx NMR suite software (Chenomx, Inc., Edmonton, AB, Canada). The performances of those models were assessed using Leave-One-Out Cross-Validation (LOOCV) with the goodness of fit (R^2^) and the ability of prediction (Q^2^) metrics. A permutation test was used to assess that the results were not obtained by chance, showed by pQ^2^ values (level of significance between the performance of a computed model and random ones).

The statistical differences in VIP between groups were computed by repeated Wilcoxon tests, and *p*-values were adjusted using the False Discovery Rate (FDR) algorithm from Benjamini and Hochberg [30].

## 3. Results

### 3.1. Baseline Characteristics

To address the primary objective of this study, namely, to characterize metabolic effects associated with respiratory illness and distinguish them from those induced by altitude training, three targeted datasets were designed and analyzed. This approach aimed to isolate infection-specific metabolic responses while accounting for confounding physiological changes induced by hypoxia and training load. The first dataset (DS1) focused on identifying illness-associated metabolic alterations by comparing urine samples collected on the day athletes reported respiratory symptoms with their corresponding baseline samples from the first day of the training camp, prior to any symptom onset. The second dataset (DS2) aimed to characterize acute metabolic responses to altitude exposure and training by comparing samples from asymptomatic athletes collected on the first and third days. As these time points represent early hypoxic exposure in the absence of illness, DS2 served as a control for evaluating short-term altitude-induced changes. The third dataset (DS3) focused on the metabolic impact of prolonged hypoxia and training stress by comparing samples from the first and eighth day, the latter coinciding with the average symptom onset in affected athletes. This dataset was used to distinguish long-term altitude adaptations from early pathological responses. DS1 included 18 samples (9 ill and 9 healthy controls), while DS2 and DS3 comprised 34 and 32 samples, respectively, from healthy athletes.

The PCA carried out on all the available data did not show any outliers. Similarly, the PCA used on each of the previously described data sets did not allow for the discrimination of the investigated conditions (Figure S1).

### 3.2. Effects of Respiratory Illnesses on Metabolic Profiles (DS1)

**Figure 1:**
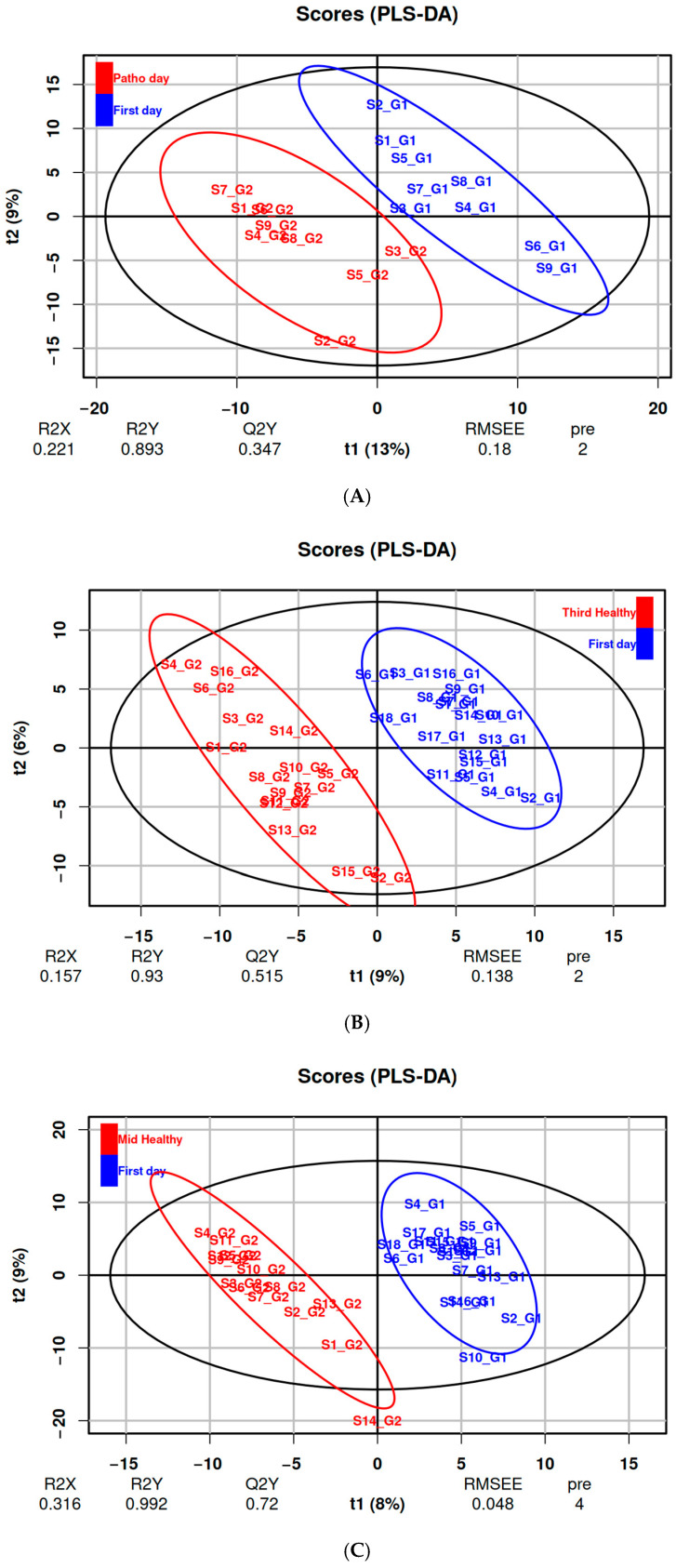
(**A**) PLS-DA score plot for the model investigating the effects of respiratory illnesses, comparing their first training camp day (blue) and the day they declared symptoms (red) (DS1). (**B**) PLS-DA score plot investigating the effects of training and hypoxia, comparing their first training camp day (blue) and the third one (red) (DS2). (**C**) PLS-DA score plot investigating the effects of training and hypoxia, comparing their first training camp day (blue) and the eighth one (red) (DS3).

In the analysis of metabolic alterations associated with respiratory illnesses, PLS-DA effectively discriminated between groups based on predefined class labels. The first two components of the model explained 13% and 9% of the total variance, respectively with cross-validation yielding an R^2^ of 0.81 and a Q^2^ of 0.36 (Figure 1A). Model validity was supported by a permutation test, indicating statistical significance with a pQ^2^-value < 0.05 (Figure S2A).

Among the most influential variables (VIP scores, *p* < 0.05), metabolites such as kynurenine, *N*-methylnicotinamide, pyroglutamate, propionate, *N*-formyltryptophan, tryptophan and glucose were identified. Two of the ten most discriminant spectral buckets remained unassigned (Table 2).

### 3.3. Effects of Altitude Training on Metabolic Profiles (DS2 & DS3)

PLS-DA discriminated metabolic profiles associated with altitude training in both datasets (Figure 1B,C), identifying several key VIPs (Table 3). The models demonstrated robust performance, with R^2^ values of 0.93 and 0.99 and Q^2^ values of 0.51 and 0.72 for the DS2 (day one vs. day three) and DS3 (day one vs. day eight) comparisons, respectively. The statistical significance of the models was confirmed by a permutation test, with pQ^2^-value < 0.05 (Figure S2B,C).

The most significant VIPs (all *p* < 0.001) included trigonelline, 3-hydroxyphenylacetate, glutamate, creatine, citrate, urea, o-hydroxyhippurate, creatinine, hippurate and alanine. Additionally, seven spectral buckets, corresponding to four unidentified metabolites, were significantly impacted but remain unassigned (Table 3).

**Figure 2:**
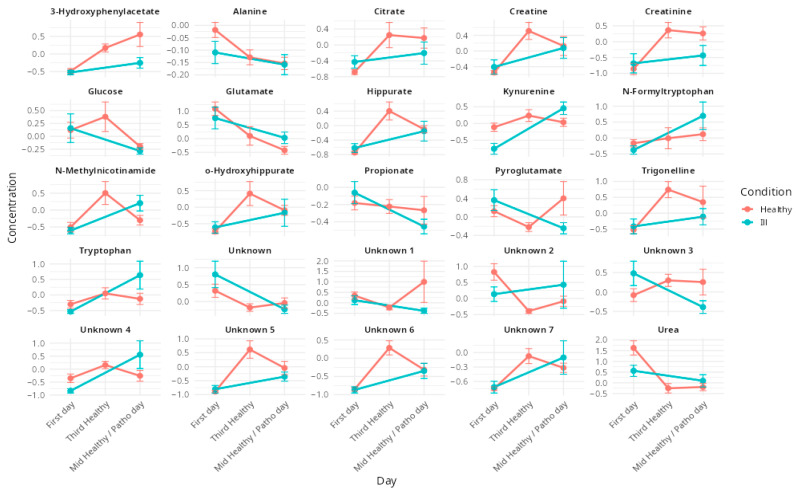
Mean metabolite concentration over time for healthy (red line) and ill (blue line) individuals. Each subplot corresponds to a specific metabolite, with error bars indicating the standard error of the mean, showing their evolution between the first training camp day (First day), the third training camp day (Third Healthy) and the eighth day or the day of symptoms occurrence (Mid Healthy/Patho day). Differences in trends between conditions highlight potential biomarkers.

The temporal evolution of these VIPs is illustrated in Figure 2, showing their evolution during the training camp and comparing evolutions in healthy and in ill athletes.

A representative 1D ^1^H NMR spectrum of urine athletes is annotated with these metabolites in Supplementary Figure S3.

## 4. Discussion

This study aimed to characterize the metabolic signatures of respiratory illnesses in elite rowers undergoing hypoxic exposure, and to differentiate these signatures from overlapping metabolic adaptations induced by altitude training. By comparing symptomatic athletes (DS1) with asymptomatic athletes undergoing altitude training (DS2 and DS3), we identified illness-specific metabolic alterations that are distinct from those associated with hypoxia-induced physiological adaptation.

### 4.1. Metabolic Impact of Altitude Training in Elite Rowers

Our findings indicate that the altitude training camp environment significantly affected urinary concentrations of several metabolites, including creatine, creatinine, citrate, trigonelline, alanine, urea, glutamate, hippurate, 3-hydroxyphenylacetate, and o-hydroxyhippurate. These metabolites have been previously associated with physiological responses to hypoxia and/or endurance exercise, supporting the notion that their modulation is consistent with established metabolic adaptations to altitude training. In particular, creatine and creatinine, well-recognized markers of muscle metabolism and energy turnover, exhibited increased concentrations over time, especially during the initial phase of hypoxic exposure (Table 3). These changes are indicative of elevated physiological stress and muscle remodeling triggered by training at altitude, in agreement with prior observations in hypoxia-exposed athletes [31]. Notably, creatine has also been proposed as a potential biomarker for acute mountain sickness reinforcing its relevance in hypoxic conditions [32].

Citrate, a key intermediate of the tricarboxylic acid cycle, also showed elevated levels, suggesting enhanced mitochondrial activity and oxidative metabolism. This adaptation is critical for maintaining ATP synthesis efficiency under reduced oxygen availability [33]. Alanine, involved in the glucose–alanine cycle, facilitates nitrogen transport and metabolic buffering between muscle and liver [34]. Its observed decrease may reflect a metabolic shift toward alternative energy pathways to support endurance performance under hypoxic stress.

Conversely, reductions in glutamate and urea concentrations were observed. Glutamate, central to nitrogen metabolism and neurotransmission, along with urea, the end-product of amino acid catabolism, may indicate decreased transamination rates or redistribution of nitrogen for anabolic processes such as tissue repair. This pattern aligns with previous evidence of hypoxia-induced reductions in glutamate levels [35], and suggests a metabolic reorganization to accommodate the sustained training load in a low-oxygen environment.

Beyond energy and nitrogen metabolism, altitude exposure also appeared to influence gut microbiota-related metabolism and oxidative stress responses. The elevation of hippurate and 3-hydroxyphenylacetate suggests gut microbial involvement and adaptive responses to oxidative stress. Hippurate is a known product of microbial metabolism and polyphenol degradation, and its increased presence may reflect training-induced changes in microbiota composition [36,37]. Similarly, 3-hydroxyphenylacetate, a downstream metabolite in phenylalanine and tyrosine pathways, has been associated with oxidative stress [38], and its rise during training at altitude may indicate increased free radical production from mitochondrial stress.

Additionally, elevated o-hydroxyhippurate levels, a gut- and liver-derived conjugate, may reflect enhanced detoxification processes aimed at maintaining metabolic homeostasis in response to intensified physiological demands.

Together, these results suggest that creatine and creatinine could serve as useful markers of muscle stress and recovery status during altitude training [31,32]. Similarly, alterations in glutamate and urea could provide insights into nitrogen handling and protein catabolism, potentially guiding training load management [35,39]. The modulation of gut microbiota-associated metabolites, such as hippurate and 3-hydroxyphenylacetate, further highlights the link between hypoxia, metabolism and gut health, suggesting that tailored nutritional strategies may support metabolic resilience and recovery [36,37,38].

Finally, trigonelline was identified as a VIP in our models and has also been reported in previous studies on endurance athletes. However, its levels are most likely influenced by coffee intake [40], and should therefore be interpreted with caution.

These observations are in line with previous metabolomic studies examining the impact of endurance exercise [41] and hypoxic [42] exposure on systemic metabolism. Pechlivanis et al. [24] reported exercise-induced alterations in urinary metabolites related to energy production and amino acid metabolism, while Lewis et al. [43] identified shifts in tricarboxylic acid cycle intermediates and markers of oxidative stress following acute physical exertion. Together, these studies support the relevance of the metabolic adaptations identified in the present work, emphasizing the interplay between energy metabolism, nitrogen balance, and gut microbial activity during prolonged exercise under hypoxic conditions.

### 4.2. Respiratory Pathologies and Their Metabolic Impact During an Altitude Training Camp

The metabolic signatures associated with respiratory illnesses observed in this study, mainly COVID-19, closely align with previously reported urinary profiles [44]. Significant disorders were identified across multiple metabolic pathways, notably those related to immune function, energy metabolism and gut microbiota activity.

Key alterations were observed in the kynurenine pathway, which plays a central role in immune regulation and inflammatory responses. Elevated concentrations of kynurenine and tryptophan in symptomatic athletes are consistent with inflammation-driven activation of tryptophan metabolism, a hallmark feature of COVID-19 and other viral infections [45]. In addition, *N*-formyltryptophan, a derivative of tryptophan, was identified as a discriminant metabolite in symptomatic individuals. This compound is implicated in oxidative stress signaling and immune modulation, further supporting the notion that respiratory infections trigger a shift toward pro-inflammatory and immunoactive metabolic states.

Beyond immune activation, the data revealed metabolic disruptions associated with cellular energy production and redox balance. *N*-methylnicotinamide, a downstream product of niacin metabolism and a key intermediate in nicotinamide adenine dinucleotide (NAD^+^) biosynthesis, was significantly elevated in symptomatic athletes. This increase likely reflects enhanced NAD^+^ turnover and oxidative stress, both characteristic of infection-related metabolic disturbances. Similar alterations in NAD^+^ metabolism have been linked to immune dysfunction and disease severity in hospitalized COVID-19 patients [46]. Moreover, pyroglutamate, a metabolite involved in the glutathione cycle and crucial for maintaining antioxidant defense was found to be decreased. Reduced pyroglutamate levels may indicate impaired glutathione homeostasis, a known consequence of systemic inflammation and oxidative stress in viral infections, including COVID-19 [47,48].

Lastly, the detection of altered levels of propionate, a short-chain fatty acid produced by the gut microbiota through the succinate pathway, points to gut immune axis involvement in the metabolic response to infection. Propionate is known to modulate immune responses and maintain intestinal barrier function. Its decrease in symptomatic athletes, compared to stable levels in healthy controls, suggests that the observed changes are more likely infection-related than diet-dependent. This is consistent with previous reports showing reduced fecal propionate concentrations in COVID-19 patients, indicative of gut dysbiosis and its contribution to systemic inflammation [49]. Altogether, these findings support the concept that respiratory illnesses, including COVID-19, elicit coordinated metabolic responses involving immune activation, oxidative stress and microbial dysregulation.

### 4.3. The Complex Interactions of Pathologies and Hypoxia

The metabolic disruptions observed in symptomatic athletes were distinct from those induced by altitude training alone. While the hypoxic environment and physical stress associated with altitude training promote beneficial adaptations that enhance oxygen utilization and energy metabolism [50], respiratory infections appear to disrupt these processes, leading to complex metabolic interactions that can negatively impact performance. Both altitude training and infection influence key physiological domains, namely immune function, oxidative stress and energy metabolism, making it challenging to differentiate between adaptive and pathological metabolic responses. Notably, all VIP metabolites were altered under both conditions (Figure 2), with group separation appearing to rely primarily on the magnitude of these changes rather than their direction.

Among the discriminant metabolites, the kynurenine pathway emerged as a key point of overlap, as it is activated both by hypoxia-induced inflammatory processes [51,52] and by immune responses to infection [45]. Elevated kynurenine and tryptophan levels in symptomatic athletes, compared to healthy ones, suggest that respiratory illness exacerbates immune activation, potentially delaying recovery. Although altitude exposure may also modulate this pathway, the absence of significant changes in kynurenine and tryptophan levels among healthy athletes after eight days at altitude indicates that the observed increases are more likely attributable to infection rather than hypoxia-driven adaptation. Similarly, *N*-methylnicotinamide, a metabolite linked to NAD^+^ turnover, was significantly elevated in symptomatic individuals, reflecting increased oxidative stress and mitochondrial strain. This dysregulation may counteract beneficial hypoxic adaptations and compromise the intended outcomes of altitude training.

Energy metabolism was also affected by both conditions. Creatine and creatinine, markers of muscle workload and adaptation, increased as expected during altitude exposure [31]. However, in the context of infection, the metabolic shift may favor catabolism over recovery, thereby impeding performance gains. Glutamate, a central amino acid in nitrogen balance, showed opposing trends in infected versus healthy athletes, further emphasizing the divergent metabolic trajectories between adaptive and pathological responses. At the level of the gut microbiota, symptomatic athletes exhibited a reduction in propionate, a short-chain fatty acid produced by gut bacteria, suggesting inflection-induced dysbiosis. In contrast, propionate levels remained stable in healthy athletes, supporting energy production and recovery processes [53].

These findings have important implications for athlete monitoring and training preparation. The additional metabolic strain of illness during altitude exposure may prolong recovery, interfere with adaptive processes and increase the risk of overtraining. Metabolic monitoring could provide a valuable tool to differentiate between normal physiological adaptation and early signs of illness, thereby enabling more precise adjustments to training loads. Moreover, ensuring sufficient recovery before altitude exposure, along with targeted nutritional strategies, such as niacin supplementation to support NAD^+^ metabolism or probiotic intake to stabilize the gut microbiota, may help mitigate these adverse effects of infection on training outcomes. Ultimately, a better understanding of these metabolic signatures can enhance altitude training protocols and contribute to optimizing athlete health and performance [54].

## 5. Conclusions

This study highlights the potential of metabolic profiling to differentiate physiological adaptations to altitude training from metabolic disturbances associated with respiratory illnesses in elite athletes. While both conditions elicit stress-related metabolic responses, our results reveal distinct underlying signatures, supporting the utility of metabolomics for the early detection of maladaptation or illness.

However, the findings are constrained by the relatively small sample size and the specificity of the athlete cohort, which may limit generalizability and subgroup analysis based on sex, despite identical training modalities. Additionally, the inherent complexity of real-world training environments presents challenges in fully disentangling the individual contributions of hypoxia, training load and infection.

Despite these limitations, the study opens promising perspectives for the application of metabolomics in athlete monitoring, particularly during physiologically demanding periods such as altitude training camps. Future research should focus on validating the identified biomarkers in larger, more diverse populations, characterizing their longitudinal dynamics across training and recovery phases, the differences between sexes, and assessing targeted interventions to enhance performance while safeguarding athlete health.

## Figures and Tables

**Table 1:** Anthropometric characteristics of study participants. Values are reported as mean ± standard deviation (SD), median, minimum (Min) and maximum (Max) for age, height, weight and body mass index (BMI). *p*-values are resulting from a t-test between men and women values; * statistical difference with *p* < 0.05.

	Men ♂ (n = 15) Women ♀ (n = 12)	Mean ± SD	Median	Min	Max
Age (years)	♂	26.4 ± 4.3	26.0	21.0	34.0
	♀	24.5 ± 2.5	24.0	20.0	29.0
Height (cm)	♂	191 ± 5.8 *	193.5	179.0	196.0
	♀	179.4 ± 7.8	177.0	168.0	197.0
Weight (kg)	♂	88.8 ± 7.7 *	90.0	70.0	97.0
	♀	71.5 ± 7.0	73.0	60.0	81.0
BMI	♂	24.1 ± 1.1	23.9	21.8	25.5
	♀	22.3 ± 2.4	21.9	18.0	25.4

**Table 2:** Identified VIPs’ buckets, for the model investigating pathology impacts (DS1, Healthy vs. Ill), with their corresponding metabolites. Negative mean values denote lower concentration in Ill athletes.

Buckets	Mean	SD	*p*-Value	Metabolite
B1_1239	−0.39	0.13	0.0314685	Propionate
B1_1539	−0.51	0.25	0.0625257	Unknown 1
B2_4381	−0.61	0.31	0.0244344	Pyroglutamate
B4_1758	1.22	0.34	0.0141917	Kynurenine
B4_4010	−0.87	0.45	0.0399835	Unknown 3
B4_6451	−0.44	0.65	0.4894282	Glucose
B7_2429	1.07	0.91	0.0399835	*N*-Formyltryptophan
B7_3101	1.18	1.17	0.0770053	Tryptophan
B7_3634	1.39	1.35	0.0399835	Unknown 4
B9_2733	0.81	0.41	0.0187577	*N*-Methylnicotinamide

**Table 3:** Identified VIPs’ buckets, for the model investigating hypoxia and training impacts (DS2 and 3, day 1 vs. day 3 and 8), with their corresponding metabolites. Negative mean values denote lower concentration on day 3 or 8 compared to day 1.

Buckets	Mean	SD	*p*-Value	Metabolite
B3_0612	1.22	0.22	0.0002649	Creatinine
B3_7065	−1.22	0.8	0.0000024	Unknown 2
B3_9187	1.06	0.65	0.0000103	Creatine
B5_7836	−1.88	0.53	0.0000650	Urea
B6_7557	0.67	0.2	0.0000082	3-Hydroxyphenylacetate
B8_5324	1.51	1.06	0.0000000	Unknown 5
B8_5510	1.14	0.56	0.0000000	Unknown 6
B8_5647	0.67	0.47	0.0000103	Unknown 7
B8_8263	1.27	0.47	0.0000024	Trigonelline
B1_4223	−0.14	0.04	0.0020369	Alanine
B2_3688	−1.52	0.5	0.0000039	Glutamate
B2_5771	0.86	0.68	0.0000718	Citrate
B3_4768	1.76	2.22	0.0000029	3-Hydroxyphenylacetate
B7_5531	0.65	0.28	0.0002836	Hippurate
B7_8284	0.64	0.26	0.0002358	o-Hydroxyhippurate

## Data Availability

The data presented in this study are available on reasonable request from the corresponding author due to privacy.

## Supplementary Materials

The following supporting information can be downloaded at https://www.mdpi.com/article/10.3390/metabo15060408/s1, Figure S1: (a) PCA score plot on data from subjects with symptoms comparing their first training camp day (blue) and the day they declared symptoms (red) (DS1). (b) PCA score plot on data from subjects without symptoms comparing their first training camp day (blue) and the third one (red) (DS2). (c) PCA score plot on data from subjects without symptoms comparing their first training camp day (blue) and the eighth one (red) (DS3); Figure S2: Permutation results on each data set (A: DS1; B: DS2, C: DS3). Each plot shows the distribution of R2Y (grey) and Q2 (black) values from 1000 permutations of the class labels (y-axis), plotted against the similarity between permuted and actual group labels (x-axis). The original model values (at similarity = 1) are displayed on the far right of each plot. A pQ^2^ value < 0.05 indicates that the predictive power of the model is significantly better than would be expected by chance; Figure S3: ^1^H NMR urine spectra sample (ill athlete on day 9) with the identified VIPs from the model investigating the effect of illness. The inset highlights a specific spectral region (7 to 8 ppm).

## Abbreviations

The following abbreviations are used in this manuscript:

^1^H NMR	Proton Nuclear Magnetic Resonance
TMSP	3-(trimethylsilyl)propionic-2,2,3,3-d4 acid sodium salt
SD	Standard deviation
PCA	Principal Component Analysis
PC	Principal Component
PLS-DA	Partial Least-Square Discriminant Analysis
VIP	Variable Importance in Projection
LOOCV	Leave-One-Out Cross-Validation
R2	Goodness of fit
Q2	Ability of prediction
FDR	False Discovery Rate
