# Supplementary material for: Metabolomics Signatures of a Respiratory Tract Infection During an Altitude Training Camp in Elite Rowers
Source: Metabolites. 2025 Jun 17;15(6):408. doi: 10.3390/metabo15060408 (PMC12195379; doi:10.3390/metabo15060408)
Supplement: Supplementary file 1 [file metabolites-15-00408-s001.zip › metabolites-3691895-supplementary.pdf]

Supplementary material (Metabolomics signatures of a respiratory tract infection during an altitude training camp in elite rowers)

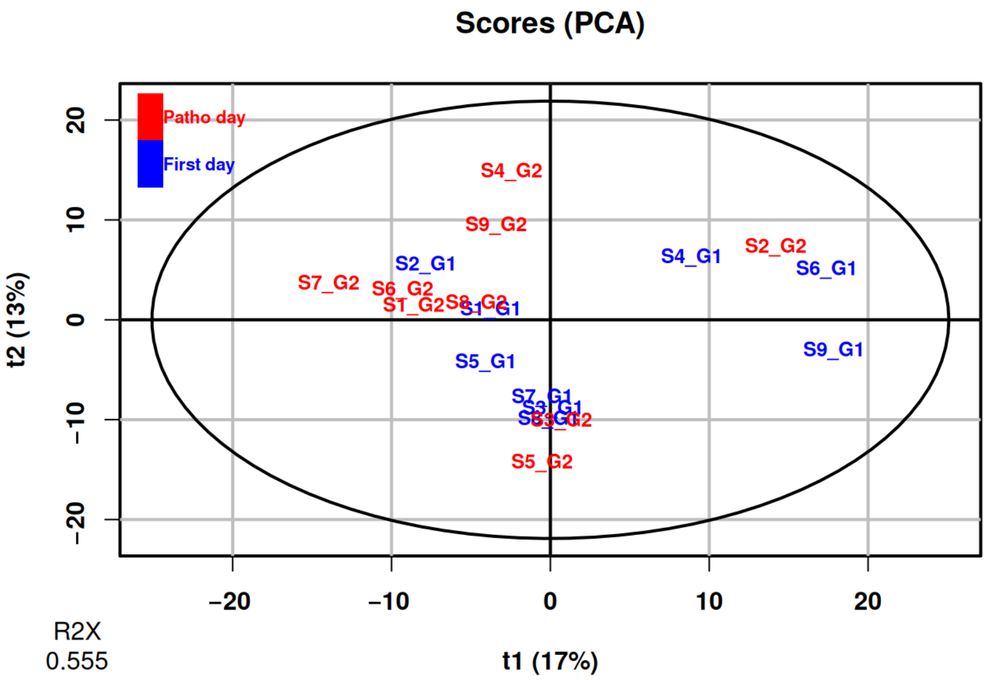

a

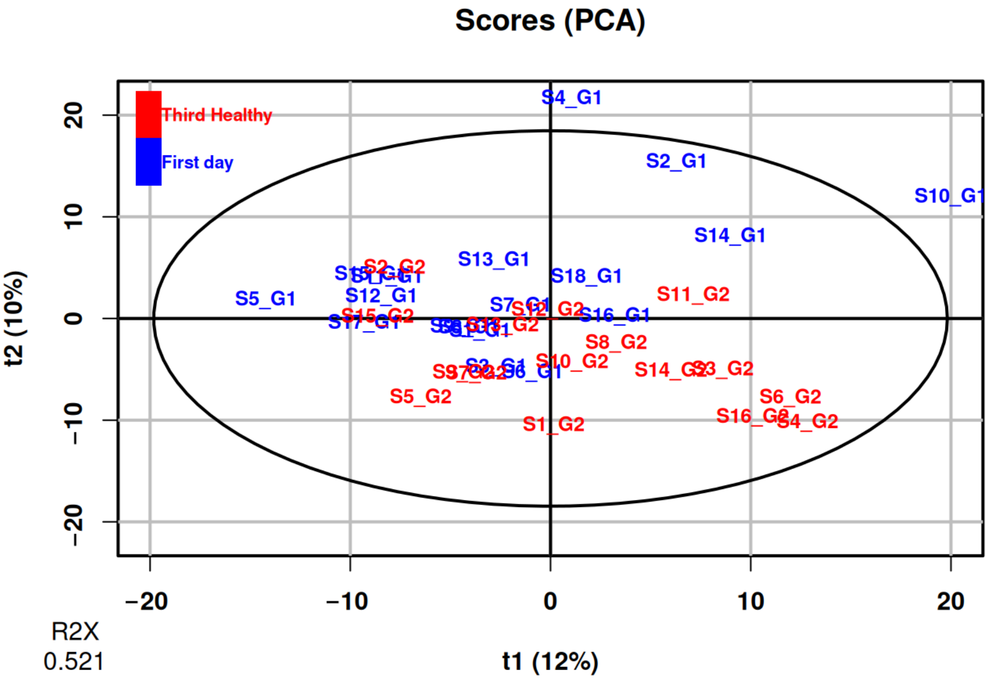

b

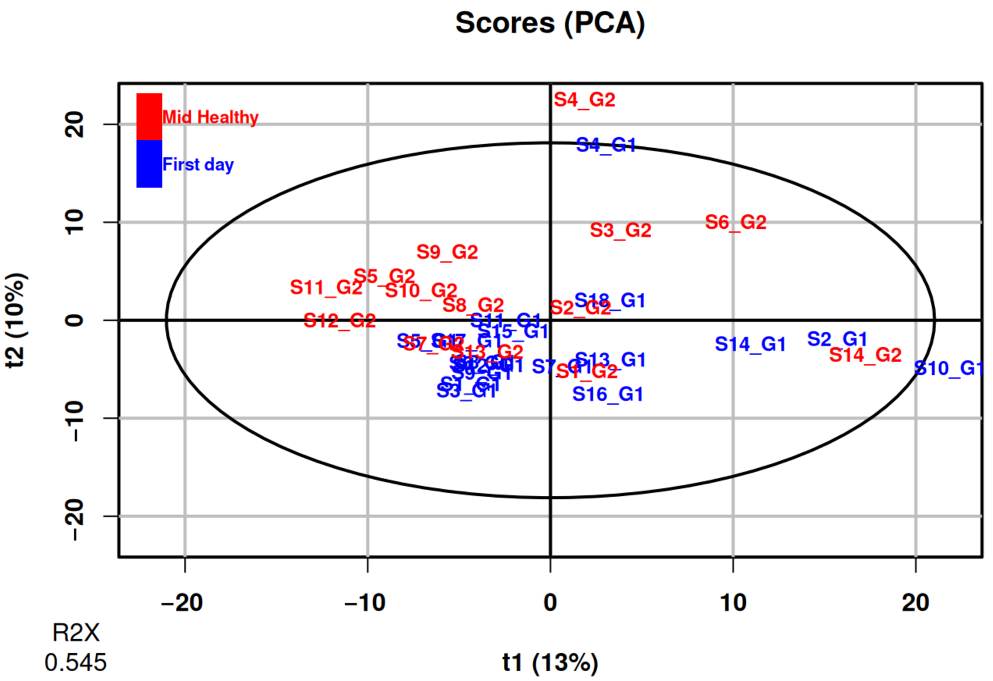

c

Figure S1: a) PCA score plot on data from subjects with symptoms comparing their first training camp day (blue) and the day they declared symptoms (red) (DS1). **b)** PCA score plot on data from subjects without symptoms comparing their first training camp day (blue) and the third one (red) (DS2). **c)** PCA score plot on data from subjects without symptoms comparing their first training camp day (blue) and the eighth one (red) (DS3).

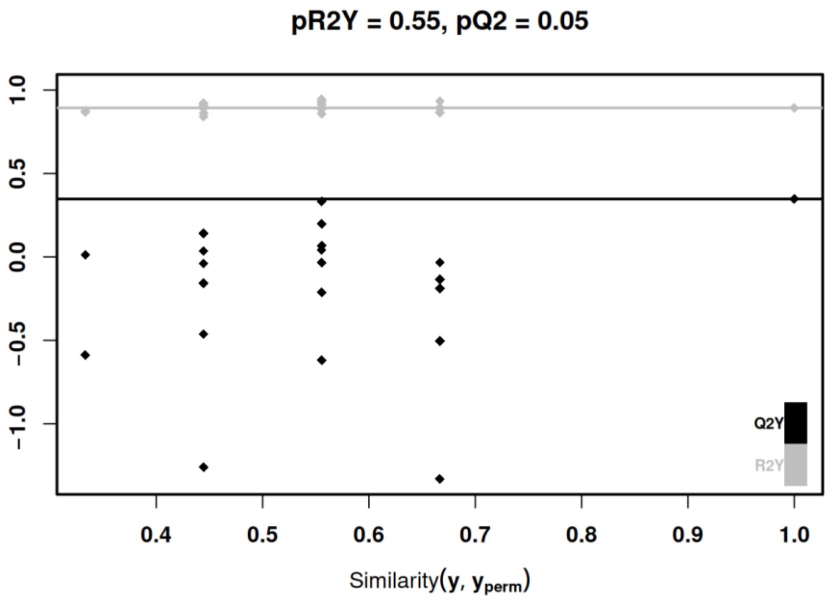
pR2Y = 0.55, pQ2 = 0.05
1.0
0.5
0.0
−0.5
−1.0
Q2Y
R2Y
0.4
0.5
0.6
0.7
0.8
0.9
1.0
Similarity(y, yperm)

a

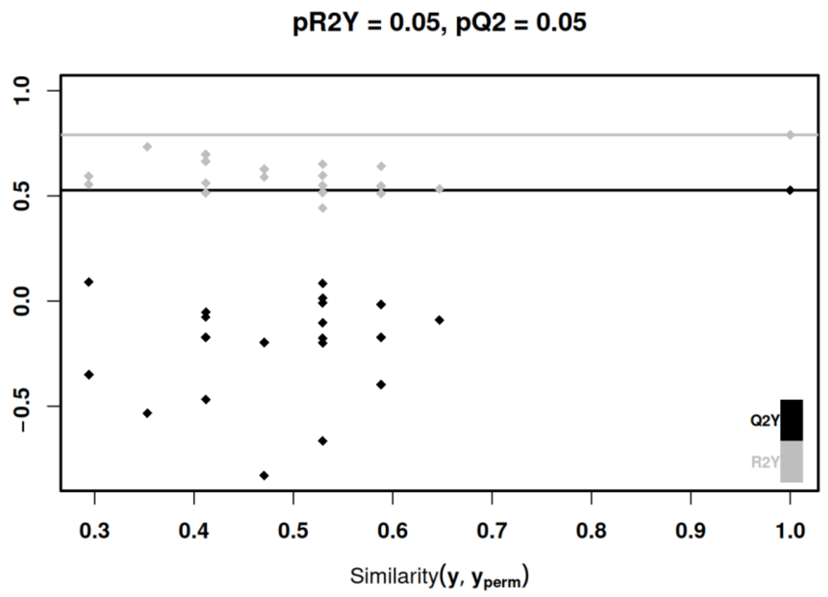
pR2Y = 0.05, pQ2 = 0.05
1.0
0.5
0.0
−0.5
Q2Y
R2Y
0.3
0.4
0.5
0.6
0.7
0.8
0.9
1.0
Similarity(y, yperm)

b

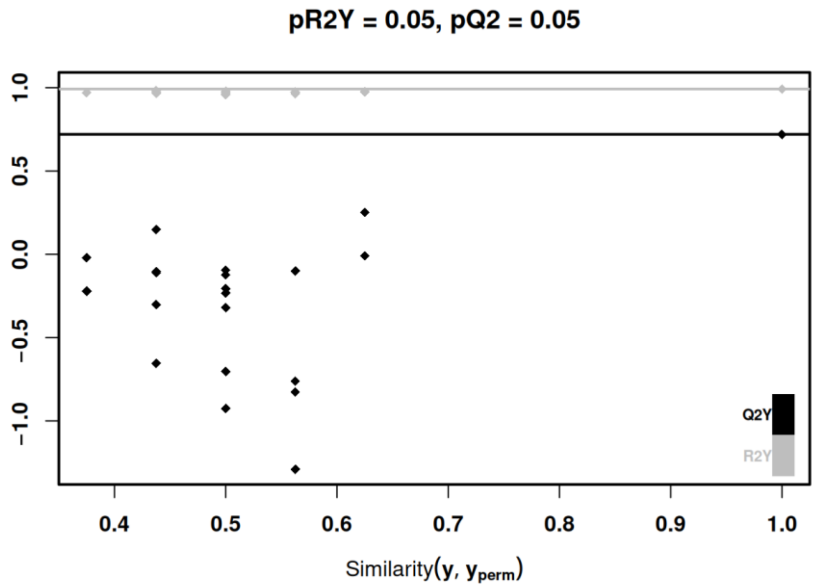

c

Figure S2: Permutation results on each data set (**a:** DS1; **b:** DS2, **c:** DS3). Each plot shows the distribution of R2Y (grey) and Q2 (black) values from 1000 permutations of the class labels (y-axis), plotted against the similarity between permuted and actual group labels (x-axis). The original model values (at similarity = 1) are displayed on the far right of each plot. A pQ2 value < 0.05 indicates that the predictive power of the model is significantly better than would be expected by chance.

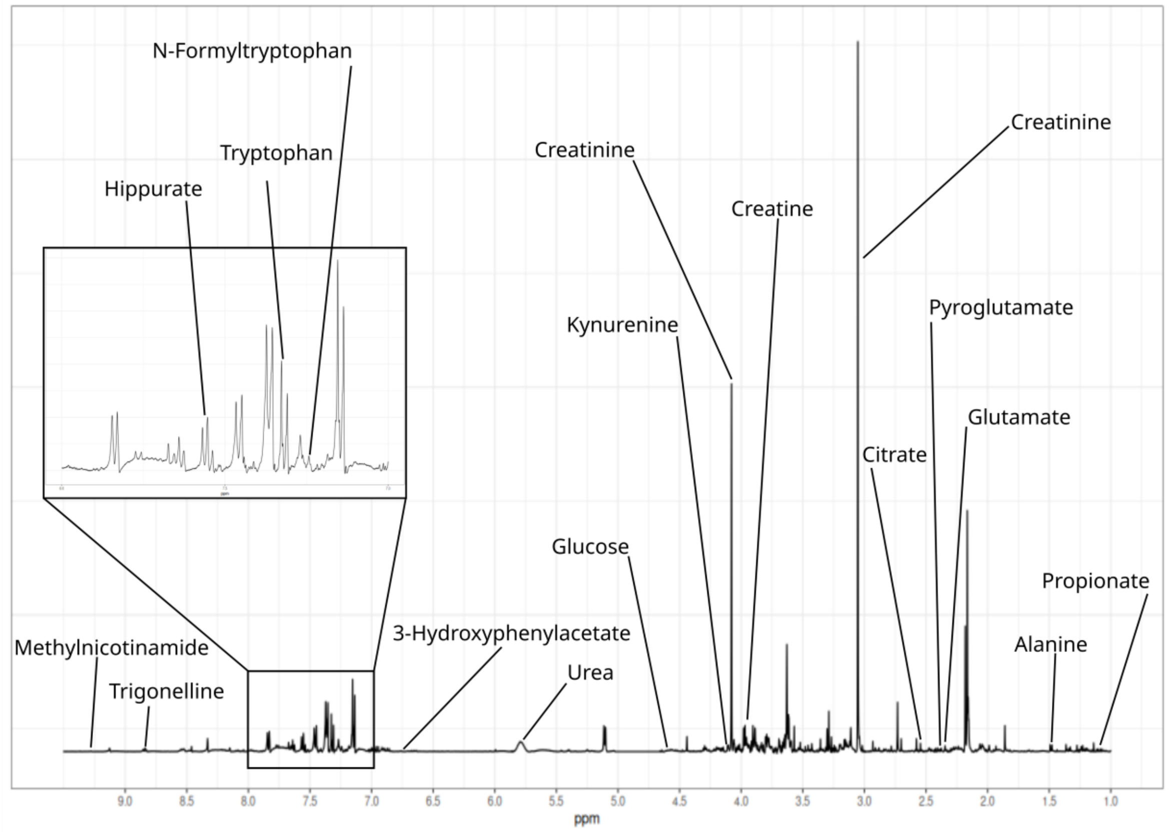

Figure S3: Representative 1D ^{1}H-NMR spectrum of athlete urine from a symptomatic athlete on day 9 with significant VIPs annotation.
